# Effectors of Th1 and Th17 cells act on astrocytes and augment their neuroinflammatory properties

**DOI:** 10.1186/s12974-017-0978-3

**Published:** 2017-10-16

**Authors:** Chittappen K. Prajeeth, Julius Kronisch, Reza Khorooshi, Benjamin Knier, Henrik Toft-Hansen, Viktoria Gudi, Stefan Floess, Jochen Huehn, Trevor Owens, Thomas Korn, Martin Stangel

**Affiliations:** 1 Clinical Neuroimmunology and Neurochemistry, Department of Neurology, Hannover Medical School, Carl-Neuberg-Str. 1, 30625 Hannover, Germany; 2 Department of Neurobiology Research, Institute of Molecular Medicine, University of Southern Denmark, Odense, Denmark; 3 Hans Christian Andersen Children’s Hospital, Odense University Hospital, Odense, Denmark; 4 Department of Clinical Immunology, Odense University Hospital, Odense, Denmark; 5 Experimental Immunology, Helmholtz Centre for Infection Research, Inhoffenstr. 7, 38124 Braunschweig, Germany; 6 Center of Systems Neuroscience, Hannover, Germany; 7 Department of Neurology, Klinikum rechts der Isar, Technische Universität München, Ismaninger Str. 22, 81675 Munich, Germany; 8 Munich Cluster for Systems Neurology (SyNergy), Munich, Germany

**Keywords:** Astrocytes, Th1, Th17

## Abstract

**Background:**

Autoreactive Th1 and Th17 cells are believed to mediate the pathology of multiple sclerosis in the central nervous system (CNS). Their interaction with microglia and astrocytes in the CNS is crucial for the regulation of the neuroinflammation. Previously, we have shown that only Th1 but not Th17 effectors activate microglia. However, it is not clear which cells are targets of Th17 effectors in the CNS.

**Methods:**

To understand the effects driven by Th17 cells in the CNS, we induced experimental autoimmune encephalomyelitis in wild-type mice and CD4^+^ T cell-specific integrin α4-deficient mice where trafficking of Th1 cells into the CNS was affected. We compared microglial and astrocyte response in the brain and spinal cord of these mice. We further treated astrocytes with supernatants from highly pure Th1 and Th17 cultures and assessed the messenger RNA expression of neurotrophic factors, cytokines and chemokines, using real-time PCR. Data obtained was analyzed using the Kruskal-Wallis test.

**Results:**

We observed in α4-deficient mice weak microglial activation but comparable astrogliosis to that of wild-type mice in the regions of the brain populated with Th17 infiltrates, suggesting that Th17 cells target astrocytes and not microglia. In vitro, in response to supernatants from Th1 and Th17 cultures, astrocytes showed altered expression of neurotrophic factors, pro-inflammatory cytokines and chemokines. Furthermore, increased expression of chemokines in Th1- and Th17-treated astrocytes enhanced recruitment of microglia and transendothelial migration of Th17 cells in vitro.

**Conclusion:**

Our results demonstrate the delicate interaction between T cell subsets and glial cells and how they communicate to mediate their effects. Effectors of Th1 act on both microglia and astrocytes whereas Th17 effectors preferentially target astrocytes to promote neuroinflammation.

**Electronic supplementary material:**

The online version of this article (10.1186/s12974-017-0978-3) contains supplementary material, which is available to authorized users.

## Background

Inflammation of the central nervous system (CNS) during multiple sclerosis (MS) or the animal model experimental autoimmune encephalomyelitis (EAE) is believed to be mediated by autoreactive T cells that are primed in the periphery and infiltrate into the CNS through the blood-brain barrier (BBB) [1, 2]. Among T cells, predominantly, IFN-γ-producing Th1 cells and IL-17-producing Th17 cells are key players in the pathogenesis of MS and EAE [3, 4]. Much of our understanding has been obtained from adoptive transfer experiments in rodent EAE models, which suggest that both Th1 and Th17 cells can mediate the disease in the CNS albeit with a varying degree of severity [5–8]. Due to the complexity of the neuroinflammatory process, there is only little knowledge available on the individual contribution of pathogenic Th1 and Th17 cells in regulating glial cell function in the CNS.

Of the CNS-resident cells, microglia and astrocytes are known to play a central role in regulating the neuroinflammatory process [9–12]. Infiltrating effector T cells are in constant crosstalk with resident glial cells, and recently, we have demonstrated that effector molecules secreted by Th1 cells but not Th17 cells influence the phenotype and function of microglia [13]. This was rather puzzling, considering the fact that Th17 cells are highly pathogenic and microglia are highly immunoreactive cell types in the CNS.

Only little is known about the crosstalk between T cells and astrocytes, the major glial cell type of the brain. Previously, astrocytes were considered to be involved in the structural framework of neural tissue while their immunoreactive properties have been underestimated. Furthermore, their intimate association with the BBB makes them one of the first glial types to encounter cells infiltrating into the CNS [14]. Subsequently, astrocyte activation regulates microglial recruitment and leukocyte trafficking in the CNS during neuroinflammatory conditions [15, 16]. In the past two decades, the contribution of astrocytes to pro- and anti-inflammatory processes in the CNS has gained prominence [17–20]. The importance of astrocytes in regulating CNS inflammation has been demonstrated by several studies which employed strategies such as ablation of reactive astrocytosis or blocking of selected receptor signaling specifically on astrocytes [10, 12, 21–23]. Upon activation, astrocytes upregulate major histocompatibility class (MHC)-I, MHC-II and co-stimulatory molecules on their surface, which highlights their ability to interact and present antigens to T cells [24–26]. Nevertheless, there is only limited knowledge about how the effector molecules released by inflammatory Th1 and Th17 cells regulate the function of astrocytes. In this study, we identified astrocytes as targets of Th17 cells in the CNS and studied the ability of effector molecules released by Th1 and Th17 cells to influence their phenotype and function.

## Methods

### Mice

C57BL/6 mice were obtained from Charles River (Sulzfeld, Germany) and housed under specific pathogen-free conditions in the central animal facility of Hannover Medical School (MHH), Germany. α4^*flox/flox*^ has been described previously [27]. *CD4 Cre* mice were obtained from The Jackson Laboratory (Bar Harbor, ME, USA). CD4^+^ T cell-conditional α4 integrin-deficient (α4^−/−^) mice were generated by crossing α4^*flox/flox*^ mice with *CD4 Cre* mice [28]. For EAE experiments, we obtained glial fibrillary acidic protein (GFAP) HSV-thymidine kinase (TK) mice on C57BL/6 background from The Jackson Laboratory (Bar Harbor, ME, USA) and the control wild-type C57BL/6 mice in this case were from Taconic (Taconic Europe, Ejby, Denmark). Animal experiments were performed according to international guidelines on the use of laboratory animals [29].

### Experimental autoimmune encephalomyelitis

EAE was induced in GFAP HSV-TK mice, α4^−/−^ mice, and control B6 mice by immunization with MOG_35–55_ peptide in complete Freund’s adjuvant, as described previously [12, 28]. Mice were immunized at two subcutaneous sites and received a total of 100 μg peptide and 200 or 250 μg adjuvant. Additionally, 15 ng/g or 200 ng pertussis toxin was administered i.p. on days 0 and 2. GFAP HSV-TK and respective controls mice were scored on a 6-point scale as follows: 0, no symptoms; 0.5, partial loss of tail tonus; 1, complete loss of tail tonus; 2, difficulty to right, 3, paresis in one or both hind legs; 4, paralysis in one or both hind legs; 5, front limb paresis; and 6, moribund. All mice used in the experiments were sacrificed 7 days after the onset of clinical symptoms. Ataxic EAE in α4^−/−^ mice was scored, by four clinical subtests and categories of ledge walking, hindlimb clasp, gait ataxia, and kyphosis with a maximum of 3 points in each category, resulting in a potential maximum score of 12 points [30]. Clinical signs of classical EAE in respective wild-type controls mice were assessed as reported [31].

### Antibodies and reagents

Antibodies specific for mouse, anti-CD4 PerCP Cy5.5 (clone: RM 4.5), anti-CD8 APC (clone 53.6.7), anti-CD11c APC (clone: N418), anti-IFN-γ APC (clone: XMG1.2), anti-CD62L APC eFluor 780 (clone: MEL-14), anti-F4/80 APC (clone: BM8), anti-CD3 (unconjugated, clone: 145-2C11), anti-CD28 (unconjugated, clone: 37.51), and fixable viability dye eFluor 506 were purchased from eBioscience (Frankfurt, Germany). Antibodies to anti-CD25 APC (clone: PC61), anti-IL-17A Pacific Blue (clone: TC11-18H10.1), anti-CD11b PE (clone: M1/70), anti-B220 APC (clone RA3-6B2), and anti-IL-10 FITC (JES5-16E3) were purchased from BioLegend (San Diego, CA, USA). Unconjugated, anti-IFN-γ (clone: XMG1.2), and anti-IL-4 (clone: 11B11) and anti-IL-2 (clone JES6-1A12) were obtained from Bio X Cell (NH, USA). Recombinant murine IL-6, IL-1β, granulocyte macrophage colony-stimulating factor (GM-CSF), and porcine TGF-β1 were purchased from R&D Systems (Wiesbaden-Nordenstadt, Germany) whereas recombinant murine IFN-γ, TNF-α, and IL-12p70 were from PeproTech (Hamburg, Germany). Recombinant murine IL-17A was obtained from BioLegend (San Diego, CA, USA).

### In vitro differentiation of Th1 and Th17 cells

Naïve CD4^+^CD25^−^ cells were differentiated in vitro into Th1 and Th17 cells as previously described [13] with slight modifications. Briefly, after enrichment of CD4^+^ T cells from the spleen and lymph nodes of C57BL/6 mice using CD4^+^ T cell enrichment kit (BD Biosciences), naïve CD4^+^CD62L^hi^CD25^−^ cells were sort purified using MoFlo (Beckman Coulter) or FACSAria (BD Biosciences). Cells (5.0 × 10^5^/ml) were stimulated with plate-bound anti-CD3 (2 μg/ml) and anti-CD28 (2 μg/ml) in 12-well plates (Corning Life Science, Acton, MA, USA) in complete Iscove’s Modified Dulbecco’s Medium (IMDM, 10% FCS, 1 mM sodium pyruvate, 50 μM β-mercaptoethanol, 25 mM HEPES, and non-essential amino acids) supplemented with either Th1-polarizing factors IL-12 (20 ng/ml) and anti-IL-4 (10 μg/ml) or with Th17-polarizing factors TGF-β1 (2 ng/ml), IL-6 (30 ng/ml), TNF-α (20 ng/ml), IL-1β (10 ng/ml), anti-IL-2 (10 μg/ml), and anti-IFN-γ (10 μg/ml). After 6 days of culture, Th1 and Th17 cells were harvested and restimulated in 12-well plates coated with anti-CD3 and anti-CD28 antibodies for 6 h. Supernatants devoid of cells were collected and stored at − 80 °C until further use.

### Primary mouse mixed glial cultures

Primary cultures of mixed glial cells were prepared from brains of postnatal 1–3-day-old C57BL/6 mice as described [13]. Briefly, the brains were freed from meninges and digested enzymatically with 0.1% trypsin (Sigma-Aldrich) and 0.25% DNase (Roche, Mannheim, Germany). Single cell suspensions obtained from the digested brains were seeded into poly-l-lysine-coated T-75-mm^2^ culture flasks in complete DMEM (DMEM + l-glutamine + 4.5 g/L d-glucose; Gibco^®^, Darmstadt, Germany) supplemented with 10% fetal calf serum (FCS), 50 U/ml penicillin, and 50 μg/ml streptomycin (all from Biochrom AG, Berlin, Germany). Microglia were harvested from the confluent cultures by shaking the culture flasks at 37 °C for 40 min at 180 rpm in an orbital shaker. Remaining microglia and proliferating oligodendrocyte precursor cells were eliminated by overnight shaking at 37 °C and 170 rpm in an orbital shaker, followed by cytosine arabinoside (AraC; 100 μM; Sigma-Aldrich) treatment for 3 days. Astrocytes were harvested from the culture flasks by mild trypsinization and were replated into six-well plates at a density of 3 × 10^5^ cells per well. Confluent astrocyte cultures were shaken at 37 °C and 180 rpm in an orbital shaker for 4 h to eliminate any remaining contaminating microglia. Astrocytes obtained in this way were referred to as highly enriched as they only had < 3% of microglial contamination (CD11b^+^ cells).

### Stimulation of astrocytes with Th1- and Th17-derived supernatants and recombinant cytokines

Confluent astrocyte cultures as obtained in the above procedure were treated for 16 h with Th1- or Th17-derived culture supernatants diluted with an equal volume of complete DMEM. In all experiments, medium controls refer to T cell culture medium (complete IMDM) collected, frozen, and diluted with an equal volume of complete DMEM just before the treatment of astrocytes. In some experiments, astrocytes were treated for 16 h with recombinant murine IFN-γ (50 ng/ml), TNF-α (10 ng/ml), GM-CSF (5 ng/ml), and IL-17A (50 ng/ml) either individually or in combination with others.

### Reverse transcription polymerase chain reaction

RNA was isolated from the cell pellet using the RNeasy Mini Kit (Qiagen) according to the manufacturer’s instructions. Equal amounts of RNA (750–1000 ng) were subsequently transcribed into complementary DNA (cDNA) with the High-Capacity cDNA Reverse Transcription Kit (No. 4368814; Applied Biosystems^®^; Life Technologies GmbH, Darmstadt, Germany). For gene expression analysis, quantitative real-time PCR was performed using the StepOne™ Real-Time PCR System and appropriate TaqMan probes (Applied Biosystems, see Additional file 1). The ΔΔCt method was applied to determine differences in the expression between astrocytes treated with medium and Th1 and Th17 supernatants. For determination of expression of retinoic acid orphan receptor c (*Rorc*) messenger RNA (mRNA) from the spinal cord, 1 μg of RNA was used for cDNA synthesis. Changes in mRNA expression levels were calculated after normalization to hypoxanthine phosphoribosyltransferase (*Hprt1*) and glyceraldehyde 3-phosphate dehydrogenase (*Gapdh*).

### ELISA

Supernatants of astrocytes cultured in the medium and Th1 and Th17 supernatants, respectively, were collected and stored at − 80 °C until further use. MCP-1/C-C chemokine ligand 2 (CCL2) (R&D Systems), CCL20 (R&D Systems), and IL-6 (Thermo Fisher Scientific) were measured in the culture supernatants using enzyme-linked immunosorbent assay (ELISA) kits for mouse and carried out according to the manufacturer’s instructions. A standard curve was generated as instructed using the standards provided in the kit. The standard curve was calculated by a computer-generated four-parameter log (4-PL) fit curve.

### Histology

Animals were sacrificed at the peak of the disease and perfused with cold PBS followed by 4% paraformaldehyde fixation (pH 7.4). The brain and spinal cord were prepared separately, embedded in Tissue-Tek (Sakura), and cryopreserved in liquid nitrogen. Twelve-micrometer-thick coronal cortical brain, sagittal brainstem, and transverse lumbar spinal cord sections were prepared using a cryotome. For immunofluorescence, sections were thawed and air-dried and, following 5-min rehydration with PBS, were stained with primary rabbit polyclonal anti-GFAP (Dako) or polyclonal rabbit Iba1 (Wako) antibodies in 0.1% Triton PBS for 2 h at room temperature (RT). After thorough washing, sections were incubated with Alexa Fluor 488- and Alexa Fluor 555-conjugated goat-anti-rabbit secondary antibodies, respectively, in 0.1% Triton PBS for 1 h at RT. After washing, slides were mounted with DAPI in Mowiol. Images were taken using a microscope (Olympus BX41) with camera.

### Transmigration of microglia

Astrocytes at 5 × 10^4^ cells per well were plated into the lower chamber of 24-well Transwell plates and cultured until they reached confluence. Confluent astrocyte cultures were treated with medium and Th1 and Th17 supernatants (diluted with an equal volume of DMEM 10% FCS) for 12 h. Following this, the cultures were washed to remove the stimuli; fresh complete DMEM was added and incubated for additional 4 h. Microglia harvested after shaking the mixed glial cultures were added to the Transwell inserts (8.0 μm pore, 24-well format; Costar^®^, Corning, NY, USA) at 7 × 10^4^ cells per insert, and the inserts were placed into the chambers containing astrocytes. After 2 h of incubation at 37 °C, microglia on the upper side of the insert were removed by using a cotton swab. The filters were then fixed with 4% PFA, stained with DAPI (1:2000), and mounted onto a glass slide with Mowiol. The number of cells that had transmigrated to the lower side of the membrane was counted (ten random fields/filter) at × 20 magnification using an Olympus BX41 fluorescence microscope.

### Transendothelial migration of T cells

Primary C57BL/6 brain microvascular endothelial cells (BMECs) and reagents needed for culturing them were purchased from Cell Biologics (Chicago, USA). Cells used for these experiments were from passages P5–P9. Astrocytes at 3 × 10^5^ cells per well were cultured in six-well plates in complete DMEM until they reached confluence. In parallel, BMECs (2.5 × 10^5^ cells per insert) were cultured on Transwell inserts (3.0 μm pore, polycarbonate membrane, six-well format; Costar^®^, Corning, NY, USA) coated with gelatin-based coating solution until they formed a confluent monolayer. Astrocytes were treated with medium and Th1- and Th17-derived supernatants (diluted in equal volumes of complete DMEM) for 12 h and then washed thoroughly to remove the T cell supernatants. Inserts with BMEC monolayers were transferred to the chamber containing astrocytes and co-cultured for an additional period of 4 h. To study T cell migration, a total of 2 × 10^5^ Th1 and Th17 cells that were restimulated on anti-CD3/anti-CD28-coated plates for 6 h were added on top of the BMEC monolayers. After 12 h, inserts were removed; culture medium in the lower chamber containing transmigrated T cell was collected, stained with anti-mouse CD4 PerCP Cy5.5, and analyzed on FACSCalibur (BD Biosciences). Each sample was acquired completely, and the cell counts in the CD4^+^ gate were used to assess the transendothelial migration of T cells.

### Phagocytosis assay

Uptake of latex beads by microglia was measured as described [32] with slight modifications. Briefly, 7.5 × 10^4^ microglia were seeded on to astrocytes that were previously treated with medium and Th1- and Th17-derived supernatants. Th1- and Th17-derived supernatants were washed off before adding microglia. After 12 h of co-culture with astrocytes, 10^7^ Fluoresbrite™ YG carboxylate microspheres (1 μm; Polysciences, Warrington, USA) were added to the cells and incubated at 37 °C for 1 h. In parallel, cells were also incubated with beads on ice and this served as negative (4 °C) control. After thoroughly washing away non-phagocytosed beads with ice-cold PBS, cells were harvested and stained with anti-mouse CD11b APC (M1-70), and phagocytosis was measured on a flow cytometer (FACSCalibur; BD Biosciences). Shift in mean fluorescence intensity (MFI) resulting from uptake of fluorescent beads was used as a measure to assess phagocytosis. Active phagocytosis was calculated by subtracting the MFI measured in 4 °C controls from the MFI measured in samples incubated at 37 °C.

### Statistical analysis

All statistical analyses were conducted using GraphPad Prism 5.0 (GraphPad Software). All data are expressed as group mean ± SEM. All experiments were performed multiple times (*n* ≥ 4), and the data obtained was analyzed using the Kruskal-Wallis test unless otherwise stated. For analyzing EAE data, we used the Mann-Whitney test. Results were considered statistically significant at *p* < 0.05.

## Results

### Blockade of Th1 infiltration into the CNS during EAE impairs astrogliosis in the spinal cord but not in the brain

**Fig. 1:**
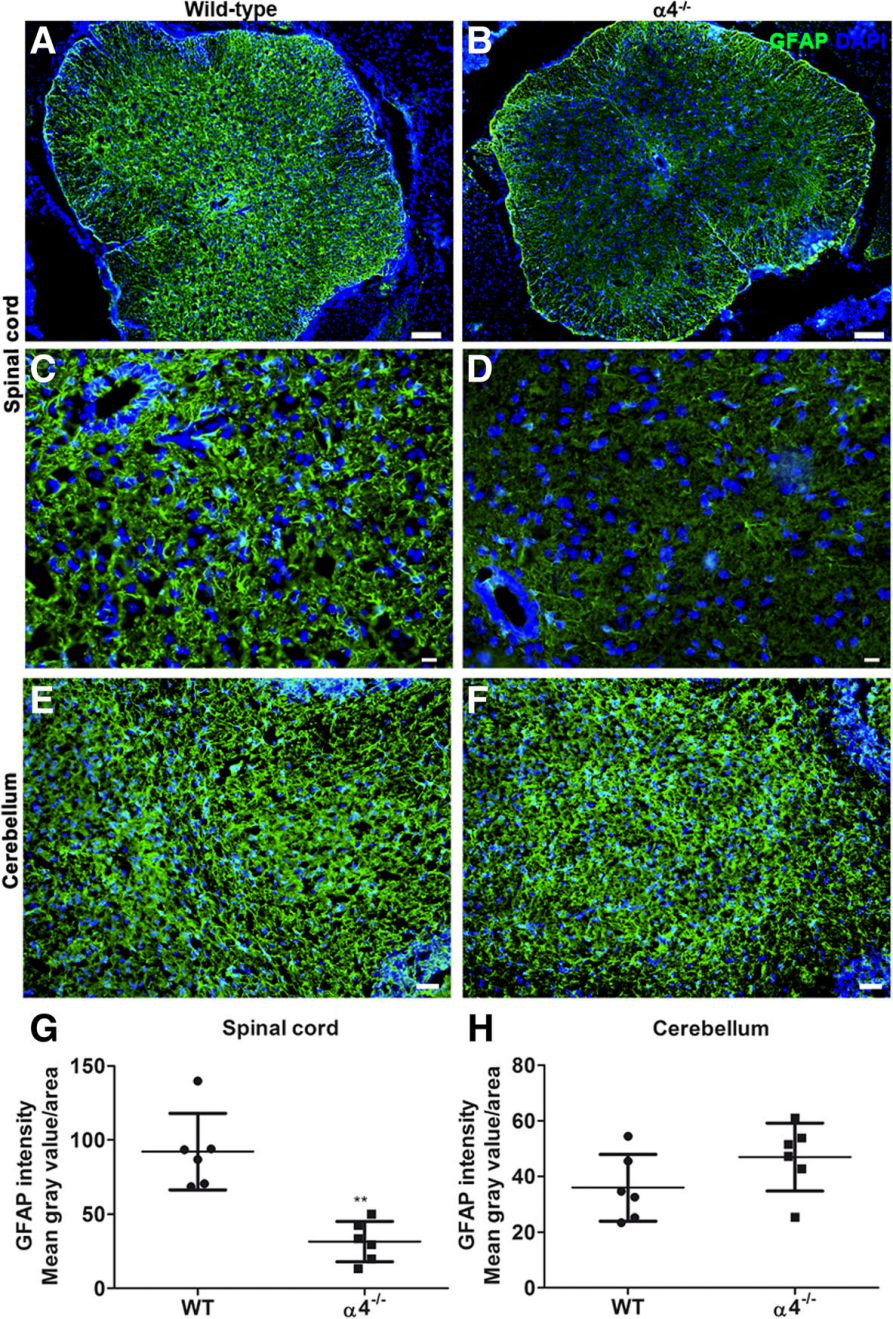
Comparison of astrogliosis between wild-type and T cell-conditional α4^−/−^ mice at the peak of EAE. Transverse lumbar spinal cord (**a**–**d**) and sagittal brainstem (**e**, **f**) sections from wild-type and T cell-conditional α4^−/−^ mice were stained for GFAP to assess astrocyte reactivity. Higher density of GFAP^+^ cells was observed in the gray matter of the spinal cord of wild-type (**a**) compared to T cell-conditional α4^−/−^ mice (**b**). Phenotypically distinct astrocytes were seen at higher magnification in wild-type (**c**) and T cell-conditional α4^−/−^ mice (**d**). Astrogliosis was comparable in the cerebellum of wild-type (**e**) and T cell-conditional α4^−/−^ mice (**f**). Bars = 200 μm (**a**, **b**), 50 μm (**c**, **d**), and 100 μm (**e**, **f**). Representative images for wild-type and α4^−/−^ mice (*n* = 6). Intensity GFAP fluorescence from the spinal cord (**g**) and cerebellum (**h**) was quantified using ImageJ software. The mean gray value of a selected area was measured after subtracting the background of 8-bit grayscale image and is plotted here. Each point represents data from one mouse. Here, we used the Mann-Whitney test for statistics; **p < 0.01

Previously, we have demonstrated that only effector molecules secreted by Th1 cells but not Th17 cells activate microglia [13]. Therefore, our aim was to identify the targets of Th17 effectors during neuroinflammation in the CNS. To address this, we employed a strategy of blocking Th1 infiltration into the CNS and studied the glial reaction in mice subjected to EAE. In an earlier study, Rothhammer et al. [28] demonstrated that blockade or deletion of integrin α4β1 (VLA-4) on CD4^+^ T lymphocytes preferentially inhibited the migration of Th1 cells into the CNS during EAE. Interestingly, blockade of Th1 migration into the CNS did not prevent the mice from developing atypical EAE, a characteristic of Th17-driven pathology (see Additional file 2), suggesting that Th17 infiltration into the CNS under these conditions was unaffected [28]. Therefore, we compared glial fibrillary acidic protein (GFAP) expression (highly expressed in reactive astrocytes) in the CNS of wild-type (WT) and T cell-conditional α4-deficient (α4^−/−^) mice subjected to EAE. GFAP staining was striking in the gray and white matter of the lumbar spinal cord of WT mice, whereas the GFAP expression was more restricted to white matter and less evident in the gray matter of α4^−/−^ mice (Fig. 1a–d). This is most likely due to the fact that Th17 infiltration into the spinal cord is reduced in the α4^−/−^ mice as reported in the previous study [28]. During EAE in α4^−/−^ mice, cellular infiltrates were found to be localized in the brainstem, cerebellum, and cerebrum [28]. Analysis of the cerebellum of WT and α4^−/−^ mice showed a similar degree of GFAP^+^ astrocytes in WT as well as α4^−/−^ mice (Fig. 1e, f). This observation allows us to assume that in the absence of Th1 cells, effectors of Th17 are equally potent to induce astrogliosis in the brain.

### Microglia are morphologically different in EAE-induced WT compared to α4^−/−^ mice

**Fig. 2:**
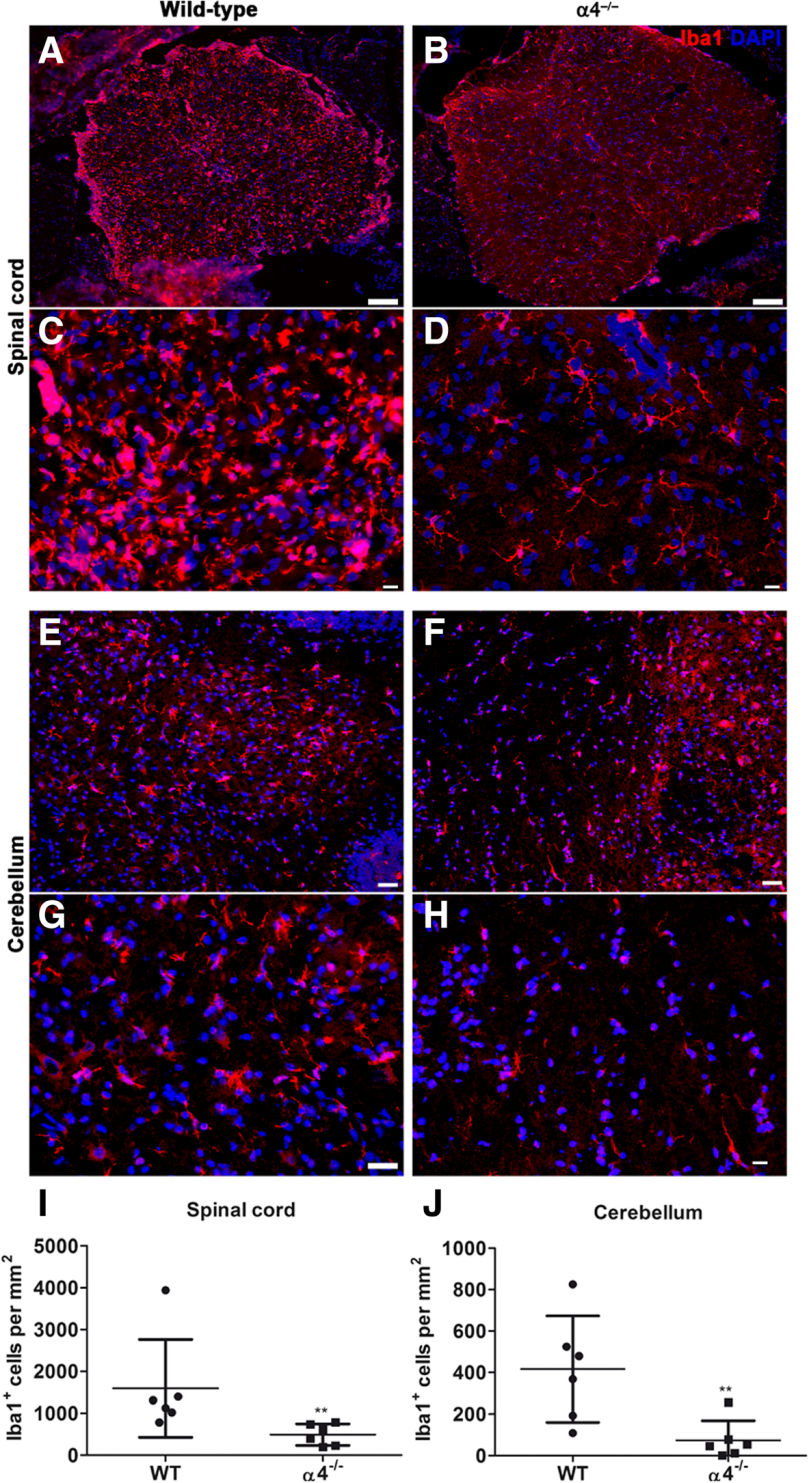
Assessment of microgliosis in wild-type and T cell-conditional α4^−/−^ mice subjected to EAE. Immunofluorescence staining for Iba1 of lumbar spinal cord sections reveals increased frequency of Iba^+^ cells in wild-type (**a**) compared to T cell-conditional α4^−/−^ mice (**b**). Note that at higher magnification, the microglia appeared morphologically different in wild-type (**c**) in comparison to T cell-conditional α4^−/−^ mice (**d**). Similar observations were made in the cerebellum of wild-type (**e**, **g**) and T cell-conditional α4^−/−^ mice (**f**, **h**). Bars = 200 μm (**a**, **b**), 50 μm (**c**, **d**, **g**, **h**), and 100 μm (**e**, **f**). Representative images for wild-type (*n* = 6) and T cell-conditional α4^−/−^ mice (*n* = 6). The density (cells/mm^2^) of Iba^+^ cells was counted in the spinal cord (**i**) and cerebellum (**j**) in identical regions of wild-type and T cell-conditional α4^−/−^ mice. Each point represents data from one mouse. Here, we used the Mann-Whitney test for statistics; **p < 0.01

We analyzed microglia in the brain and lumbar spinal cord of WT and α4^−/−^ mice subjected to EAE by immunohistochemistry. Iba1 staining revealed a higher number and morphologically distinct Iba1^+^ microglia both in the brain and spinal cord of WT mice compared to α4^−/−^ mice. Microglia detected in the spinal cord (Fig. 2) and cerebellum of WT mice exhibited a hypertrophic phenotype with shorter and thicker processes, reminiscent of highly activated microglia. In contrast, Iba1^+^ microglia in α4^−/−^ mice had a more bipolar structure with fewer and finer process (Fig. 2). This is in accordance with our previous findings where we showed Th17 effectors do not have direct influence on microglia [13]. Taken together, these observations suggest that Th17 effectors are highly capable of inducing astrocyte activation but less efficient in driving microglial activation in the brain.

### Th1- but not Th17-derived factors downregulate the expression of growth factors in astrocytes

**Fig. 3:**
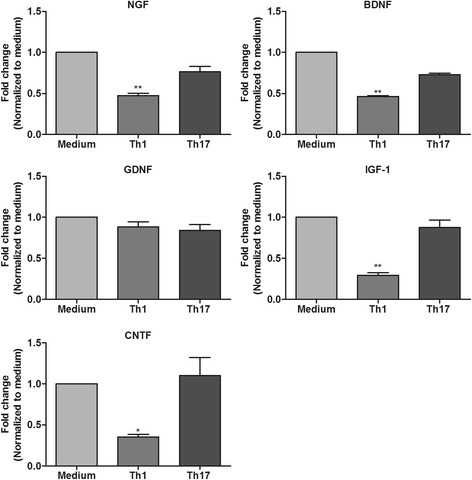
Effects of Th1- and Th17-derived factors on the expression of neurotrophic factors in astrocytes. RT-PCR analysis of RNA isolated from astrocytes treated with medium and Th1 or Th17 supernatants for nerve growth factor (NGF), brain-derived neurotrophic factor (BDNF), glial cell-derived neurotrophic factor (GDNF), ciliary neurotrophic factor (CNTF), and insulin-like growth factor 1 (IGF-1) mRNA. Changes in mRNA expression levels were calculated after normalization to hypoxanthine phosphoribosyltransferase (HPRT)1. The results are represented as fold changes which were calculated by using the ΔΔCt method (i.e., normalizing the ΔCt values obtained from subjects (Th1 or Th17 treated) to those from medium controls). The results are mean ± SEM with *p ≤ 0.05 and **p ≤ 0.01 of a minimum of four independent experiments (*n* = 4)

Having understood that astrocytes are influenced by Th17 cells, we further tested if Th1 and Th17 effectors can have direct effects on astrocytes. In our previous study, we have reported an efficient method to obtain highly pure Th1 and Th17 cells in vitro and also characterized their cytokine secretion profile [13]. The Th1 cells obtained by this method were devoid of Th17 cells and vice versa. Cytokine profiling of Th1 and Th17 cell culture supernatants revealed that Th1 cells predominantly secreted IFN-γ and GM-CSF, whereas the Th17 cells released large amounts of IL-17A and IL-17F [13]. For determining the response of astrocytes to Th1- and Th17-derived factors, we employed reverse transcription polymerase chain reaction (RT-PCR) analysis of RNA isolated from astrocytes that were treated with medium and Th1- and Th17-derived supernatants. Astrocytes are an important source of neurotrophic factors needed for the maintenance of neural tissue. Factors released by cells infiltrating from the periphery during neuroinflammation can alter their expression. Therefore, we assessed the mRNA expression of key growth factors such as nerve growth factor (NGF), brain-derived neurotrophic factor (BDNF), GDNF, ciliary neurotrophic factor (CNTF), and insulin-like growth factor (IGF)-1 in astrocytes following their treatment with Th1- and Th17-derived supernatants. Interestingly, we observed that Th1-derived supernatants caused greater than or equal to twofold downregulation in the expression of NGF, BDNF, CNTF, and IGF-1 in astrocytes. In contrast, Th17-derived supernatants did not have any influence on the expression of these neurotrophic factors (Fig. 3).

### Both Th1- and Th17-derived factors trigger a pro-inflammatory response in astrocytes

**Fig. 4:**
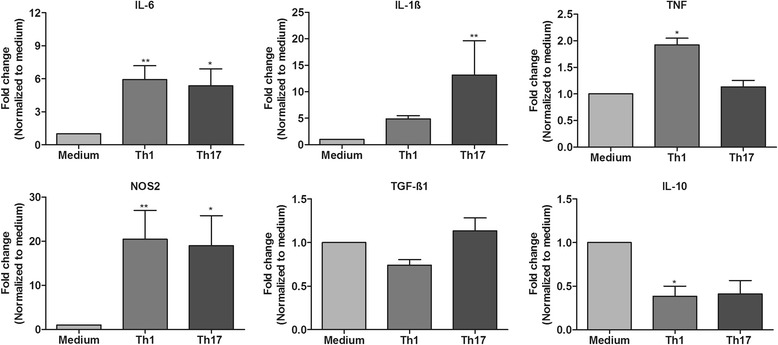
Inflammatory response of astrocytes to Th1- or Th17-derived factors. mRNA expression of pro-inflammatory (IL-1β, IL-6, TNF-α, and NOS2) and anti-inflammatory (TGF-β1 and IL-10) factors was analyzed in astrocytes exposed to medium and Th1 or Th17 supernatants. Fold change in mRNA expression in Th1- or Th17-treated astrocytes compared to medium controls was determined. The results are mean ± SEM with *p ≤ 0.05 and **p ≤ 0.01 of a minimum of four independent experiments (*n* = 4)

Apart from being a key source of growth factors, astrocytes are important mediators of inflammation in the CNS. Astrocytes produce not only key pro-inflammatory cytokines such as IL-1β and IL-6 that are neurotoxic but also some neuroprotective anti-inflammatory cytokines such as TGF-β and IL-10. We thus studied the influence of Th1- and Th17-derived factors on the inflammatory response of astrocytes. We found that in response to 16 h of treatment with Th1 and Th17 supernatants, astrocytes significantly upregulated the mRNA expression of pro-inflammatory genes such as IL-1β, IL-6, and nitric oxide synthase 2 (NOS2) (greater than twofold). TNF-α mRNA expression was enhanced by twofold in Th1-treated astrocytes and was unchanged in Th17-treated astrocytes (Fig. 4). On the other hand, although we observed no change in TGF-β1 mRNA expression, IL-10 mRNA expression was approximately twofold lower in Th1-treated (statistically significant) and Th17-treated (did not yield statistical significance) astrocytes compared to control medium-treated astrocytes (Fig. 4). In our attempt to identify the factors driving activation, we treated astrocytes with individual or a combination of recombinant cytokines based on the knowledge we gained from the multiplex data of Th1 and Th17 supernatants from our previous study [13]. Similarly, we analyzed the gene expression of pro-inflammatory chemokines and cytokines to assess astrocyte activation. These results suggest that IFN-γ remains a potent effector of Th1 cells and combines with other effectors such as TNF and GM-CSF to augment astrocyte activation. On the other hand, IL-17 alone had only minimal impact and only when given in combination with TNF to induced astrocyte activation (see Additional file 3).

### Th1- and Th17-derived factors induce chemokine expression in astrocytes

**Fig. 5:**
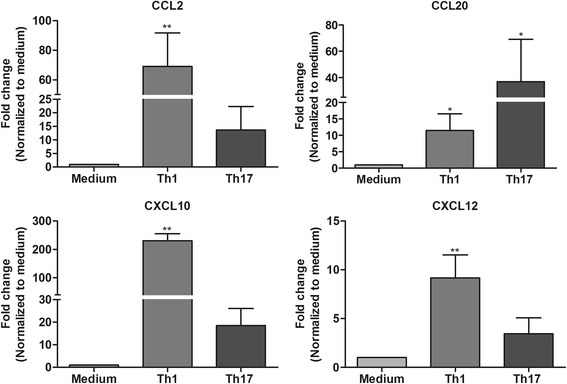
Chemokine expression profile in astrocytes exposed to medium and Th1 or Th17 supernatants. Fold changes in CCL2, CCL20, CXCL10, and CXCL12 mRNA expression in Th1- and Th17-treated astrocytes compared to medium control were measured by RT-PCR. The results are mean ± SEM with *p ≤ 0.05 and **p ≤ 0.01 of a minimum of four independent experiments (*n* = 4)

In response to an inflammatory milieu, astrocytes produce several key chemokines that assist in the recruitment of microglia and leukocytes from the periphery to the sites of inflammation. Here, we assessed the influence of Th1- and Th17-derived factors on the mRNA expression profile of chemokines in astrocytes. In general, greater than threefold upregulation of CCL2, CCL20, and C-X-C chemokine ligand 10 (CXCL10) mRNA was found in astrocytes treated with Th1 and Th17 supernatants compared to medium controls (Fig. 5). However, mRNA expression levels of CCL2 and CXCL10 were several folds higher in Th1-treated astrocytes compared to those in Th17-treated astrocytes. On the other hand, CCL20 mRNA expression was more prominently enhanced in Th17-treated astrocytes than in Th1-treated astrocytes. CXCL12 mRNA expression was significantly upregulated in astrocytes only in response to Th1- and not to Th17-derived factors (Fig. 5). In addition, enhanced expression of some of these factors (CCL2, CCL20, and IL-6) was also confirmed at the protein level (see Additional file 4).

### Enhanced migration of microglia in response to astrocytes activated by Th1- and Th17-derived factors

**Fig. 6:**
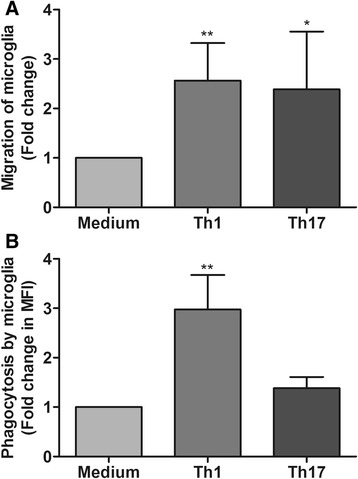
Response of microglia towards Th1- or Th17-treated astrocytes. **a** Migration of microglia from the upper side of the Transwell membrane to the lower side in response to medium and Th1- or Th17-treated astrocytes was assessed by transmigration assay performed with microglia in the upper chamber and astrocytes plated in the lower chamber of a Transwell system. Fold changes in transmigration of microglia through the membrane in response to Th1- or Th17-treated astrocytes compared to medium control were measured by counting DAPI^+^ cells in 10 randomly selected fields on the lower side of the membrane. The results are representative of a minimum of five independent experiments. **b** The phagocytic ability of microglia co-cultured with medium and Th1- or Th17-treated astrocytes was measured using a latex bead uptake assay. Uptake of fluorescently labeled beads was measured by flow cytometry. The bar graphs represent fold change in phagocytosis of microglia co-cultured with Th1 or Th17 supernatant-pretreated astrocytes compared to those cultured with medium-treated astrocytes. The results are mean ± SEM with *p ≤ 0.05 and **p ≤ 0.01 of four independent experiments (*n* = 4)

Having observed that both Th1- and Th17-derived factors act on astrocytes and enhance their chemokine expression, we were interested to study if this reflects their ability to attract microglia as it has been reported [21]. Firstly, we tested the capability of astrocytes activated by Th1- and Th17-derived factors, respectively, to recruit microglia using a Transwell migration assay. Astrocytes were treated with medium and Th1- or Th17-derived supernatants. Four hours before adding microglia to the Transwell inserts, the stimuli were removed and the fresh medium was added to exclude the influence of Th1 and Th17 effectors on microglial migration. Compared to medium-treated controls, we observed greater than twofold increase in migration of microglia towards both Th1- and Th17-treated astrocytes (Fig. 6a). Secondly, we tested if microglia recruited towards Th1- or Th17-treated astrocytes displayed any changes in their phagocytic ability. For this purpose, after eliminating the Th1- and Th17-derived factors by washing, we added microglia to Th1- and Th17-treated astrocytes. After 12 h of co-culture, uptake of latex beads by microglia as a measure of their phagocytic ability was assessed by flow cytometry. Interestingly, we observed an approximately threefold increase in the phagocytic ability of microglia co-cultured with Th1-treated astrocytes, whereas phagocytosis of microglia co-cultured with Th17-treated astrocytes was unchanged (Fig. 6b).

### Th1- and Th17-activated astrocytes enhance transendothelial migration of Th17 cells

**Fig. 7:**
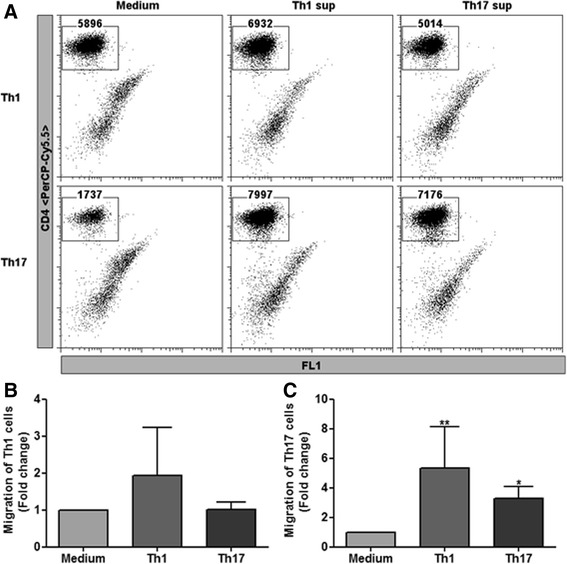
Transendothelial migration of activated Th1 or Th17 cells towards Th1- or Th17-treated astrocytes. Confluent monolayers of primary mouse brain microvascular endothelial cells (BMECs) were cultured on the upper side of Transwell inserts. These inserts were incubated for 4 h with astrocytes that were previously treated with medium and Th1 or Th17 supernatants. Following this, activated Th1 or Th17 cells were added to respective inserts and migration of T cells through the endothelial layer was determined by flow cytometry. **a** The dot plots represent the migration of Th1 cells (upper panel) and Th17 cells (lower panel) in response to medium (right) and Th1 (middle) and Th17 (left) supernatant-treated astrocytes. The numbers in each plot represent the FACS counts of cells in the CD4^+^ gate following the acquisition of the entire sample. Fold changes in migration of Th1 (**b**) and Th17 (**c**) cells in response to Th1- or Th17-treated astrocytes were calculated by normalizing to the migration observed in response to medium-treated astrocytes. The results are representative of at least four independent experiments: **b** *n* = 4 and **c** *n* = 5. All data are mean ± SEM with *p ≤ 0.05 and **p ≤ 0.01

Since chemokines such as CCL20, CXCL10, and CXCL12 are also known to play a significant role in the chemotaxis of activated T lymphocytes, we hypothesized that astrocytes activated by Th1- and Th17-derived factors might play a role in the recruitment of a second wave of Th1 and Th17 cells through the BBB. To address this, we employed a transendothelial migration assay, where migration of Th1 and Th17 cells towards activated astrocytes through a semi-compact endothelial monolayer cultured on Transwell inserts was assessed. Transwell inserts were seeded with mouse primary brain microvascular endothelial cells (BMECs) and cultured until they reached confluence. These inserts containing BMEC monolayers were transferred to six-well plates containing astrocytes that were previously treated with medium and Th1- or Th17-derived supernatants. Following anti-CD3/anti-CD28 restimulation, polarized Th1 and Th17 cells were added to the Transwell inserts and their migration towards astrocytes was measured by flow cytometry after 12 h of incubation. Intriguingly, we found activated Th1 cells transmigrated through the endothelial layer with equal efficiency towards medium and Th1- and Th17-treated astrocytes (Fig. 7a). In contrast, transendothelial migration of Th17 cells was poor in response to medium-treated astrocytes and was highly enhanced by up to threefold in response to Th1- and Th17-treated astrocytes (Fig. 7b, c).

These results were further supported by the findings from our EAE model where we compared the expression of *Rorc* mRNA between wild-type and astrocyte-depleted mice. Considering that *Rorc* expression is characteristic to infiltrating immune cells, we used *Rorc* mRNA expression to assess the amount of Th17 cells in the CNS [33]. In a previous study, we used transgenic mice (GFAP thymidine kinase (TK)) expressing TK under the GFAP promoter and depleted reactive astrocytes at day 7 after the onset of EAE by injecting ganciclovir (GCV). It was found that depletion of astrocytes resulted in enhanced infiltration of myeloid cells and subsequent enhancement of disease severity [12]. Strikingly, in mice with established EAE, astrocyte depletion showed lower *Rorc* mRNA expression in the spinal cord compared to that of non-depleted mice (see Additional file 5), hence hinting that astrocyte activation is crucial for the recruitment of Th17 cells into the CNS.

## Discussion

Previously, we have demonstrated that only effector molecules released by Th1 cells had direct influence on microglia, whereas effector molecules of Th17 cells show no direct effects on microglia [13]. In this study, we identified astrocytes as one of the targets of Th17 effectors. We observed that during EAE, infiltration of Th17 cells alone was sufficient to induce astrogliosis in the brain. Furthermore, we could demonstrate that factors derived from Th1 and Th17 cells acted on astrocytes and triggered a pro-inflammatory cytokine and chemokine response that assisted the recruitment of microglia and transendothelial migration of Th17 cells.

The current knowledge on Th1 and Th17 cells in MS pathogenesis has come mainly from EAE models where individual antigen-specific Th1 and Th17 cells were adoptively transferred into the mice. However, plasticity associated with adoptively transferred T cells is a major limitation in understanding the contributions of specific effectors in driving the neuroinflammation [34–36]. Migration of effector Th1 and Th17 cells into the CNS is assisted by a distinct set of chemokine receptors and integrins. While the integrin VLA4 (α4β1) is indispensable for Th1 migration, Th17 cells most likely depend on C-C chemokine receptor 6 (CCR6) and LFA-1 [28, 37]. Earlier work has shown that interfering with specific integrins on CD4^+^ T lymphocytes can modulate CNS infiltration of Th1 and Th17 cells [28]. Conditional knockout of α4 integrin in CD4 T lymphocytes (α4^−/−^) causes an atypical EAE in mice with predominant infiltration of Th17 cells and not Th1 cells into the brainstem, cerebellum, and forebrain [28]. Assessment of glial reactions in wild-type and α4^−/−^ mice that were subjected to EAE provided us the information of potential targets of Th17 cells in the CNS. Here, astrogliosis and microgliosis were more pronounced in the lumbar spinal cord sections of wild-type mice and less prominent in α4^−/−^ mice. This could be explained by the fact that infiltration of both Th1 and Th17 cells into the spinal cord is drastically impaired in α4^−/−^ mice [28]. In contrast, marked infiltration of Th17 cells but not Th1 cells was detected in the cerebellum and brainstem of α4^−/−^ mice [28]. Analyzing the glial reaction of the cerebellum, we detected comparable astrogliosis, whereas the density of microglia (Iba1^+^) was reduced and its phenotype was strikingly different in α4^−/−^ mice despite pronounced infiltrates of Th17 cells. These findings strongly suggest that Th17 cells and their effector molecules are capable of activating astrocytes whereas microglia are less responsive to these cells.

We further studied if effectors of Th1 and Th17 cells had any direct influence on astrocyte activation. Astrocytes are an important source of neurotrophic factors, and downregulating their expression can trigger neurodegeneration [38]. Here, we observed that Th1-derived factors significantly downregulated the expression of key neurotrophic factors like NGF, BDNF, and CNTF in astrocytes. Similarly, expression of IGF-1, a growth factor involved in the protection of neurons against oxidative stress, is also downregulated, suggesting that effectors of Th1 cells trigger neurodegeneration by suppressing the production of neurotrophic factor by astrocytes. In contrast, Th17-derived factors had no influence on the expression of neurotrophic factors in astrocytes.

Existing evidence suggests that IFN-γ and IL-17, the key cytokines secreted by Th1 and Th17 cells, respectively, are capable of regulating astrocyte function [25, 39–41]. Both Th1- and Th17-derived factors acted on astrocytes and induced a strong pro-inflammatory response where expression of IL-1β, IL-6, and NOS2 mRNA was upregulated by several folds. In addition, we observed a nearly twofold reduction in the expression of the anti-inflammatory factor IL-10 in astrocytes treated with Th1 and Th17 supernatants. Therefore, we believe that effector molecules secreted by Th1 and Th17 cells suppress an anti-inflammatory response and trigger a potent pro-inflammatory response in astrocytes. Although we have previously characterized Th1 and Th17 supernatants in terms of their cytokine profile [13], it is not known which effector molecules were responsible for driving astrocyte activation. It is noteworthy that while IFN-γ remains the major effector of Th1 cells, astrocyte activation is increased several folds when it is combined with other factors such as TNF and GM-CSF (see Additional file 3). IL-17 is the only major effector detected in our Th17 supernatants along with little amounts of TNF-α. Nevertheless, IL-17 alone had no impact on astrocytes. Interestingly, IL-17 appears to synergize with TNF-α, since we observed increased expression of IL-6 and CCL20 mRNA only when astrocytes were treated with a combination of these cytokines (see Additional file 3). Few studies in the past have reported such synergy between IL-17 and TNF-α on other cell types [42, 43].

We also observed that Th1-derived supernatants largely enhanced the mRNA expression of CCL2, CXCL10, and CXCL12, whereas CCL20 expression was highly upregulated in astrocytes treated with Th17-derived supernatants. This is an indication that effector molecules of Th1 and Th17 cells induce a selective chemokine response by astrocytes. Chemokines and their receptors act as amplifiers of neuroinflammation by assisting recruitment of immune cells from the periphery and microglia to the inflammatory foci [44]. We have previously shown that astrocytes are essential for recruitment of microglia for myelin clearance during cuprizone-induced demyelination and remyelination [21]. Similarly, we observed that astrocytes treated with Th1- or Th17-derived supernatants enhanced microglial migration towards astrocytes. Interestingly, only microglia that migrated towards Th1-treated astrocytes show enhanced phagocytosis. Microglial phagocytosis can have beneficial and detrimental effects in the CNS and can be differentially regulated by several factors [45]. One such factor, TNF-α is known to enhance the phagocytic activity of microglia [46] and we have observed that only astrocytes treated with Th1-derived supernatants show enhanced expression of TNF-α.

CCL20 is constitutively expressed by the cells in the choroid plexus and is considered to be the gateway for T cells into the CNS [37, 47]. A few studies suggest that CCR6, a receptor for CCL20, is expressed specifically on Th17 and regulatory T cells and not on Th1 cells [37, 48]. Our own experience suggests that CCR6 is also expressed on Th1 cells [49]. Nonetheless, we hypothesized that Th1 and Th17 cells might activate astrocytes and play a role in the recruitment of a second wave of Th1 and Th17 cells. We first corroborated this hypothesis using an in vitro model where we tested transendothelial migration of activated Th1 and Th17 cells towards astrocytes treated with Th1 and Th17 supernatants. Th1 cells crossed the endothelial barrier and were not dependent on the activation of the astrocytes. However, increased transendothelial migration of Th17 cells was observed only in response to astrocytes treated with either Th1- or Th17-derived supernatants.

Previously, we and others have shown that ablation of reactive astrocytes exacerbated clinical signs of EAE [12, 23]. In this model, we detected relatively lower expression of *Rorc* mRNA in the spinal cord of mice where astrocytes were depleted at the onset EAE, thus supporting our above findings that astrocytes are crucial for recruitment of Th17 cells into the CNS. Although we observe reduced Th17 signal and more severe EAE in the absence of reactive astrocytes, it must be remembered that astrocytes are active components of the BBB where they form the *glia limitans* and control the trafficking of all cells through the BBB. Compromising the BBB by depleting astrocytes leads to excess of myeloid infiltrates into the CNS, thus triggering severe neuroinflammation, and this would override the effects mediated by Th1 or Th17 cells in this model.

## Conclusion

Here, we present evidence that the effectors of Th1 and Th17 cells have distinct effects on different glial cell types. We have previously shown that only Th1-derived effectors influenced the phenotype and function of microglia whereas Th17 cells had no direct effects [13]. The data from the current study show that astrocytes are potential targets of Th17 cells in the CNS and effector molecules of both Th1 and Th17 cells had direct influence on the phenotype and function of astrocytes. From our data, we believe that Th1 cells act via both microglia and astrocytes, amplify the inflammatory response, inhibit the production of essential neurotrophic factors, and enhance the recruitment of microglia and Th17 cells by upregulation of essential chemokines. In contrast, Th17 cells only act on astrocytes and impart a pro-inflammatory phenotype to astrocytes. We believe that this ability of Th17-derived factors to act on astrocytes and not on microglia might be due to the difference in the expression of receptors or signaling molecules required for sensing Th17-derived effector molecules. An indirect evidence for this speculation comes from the work of Kang et al. who demonstrated that ablation of IL-17-induced Act1 signaling on astrocytes ameliorates EAE, whereas ablation of this signaling molecule in microglia or macrophages has no influence on the course of EAE [10]. Our results demonstrate the delicate interaction between T cell subsets and glial cells and how they communicate to mediate their effects.

## Additional files

Additional file 1: TaqMan probes used for RT-PCR analysis. (DOCX 11 kb)

Additional file 2: Clinical course of WT and α4^−/−^ mice. EAE severity was monitored in WT and α4^−/−^ mice following immunization with MOG_35–55_ in CFA. WT mice showed the signs of classical EAE whereas α4^−/−^ mice had an ataxic EAE syndrome. Data is represented as mean clinical score + SEM (*n* = 6). (TIFF 419 kb)

Additional file 3: Astrocyte response to effector cytokines. Astrocytes treated with recombinant cytokines for 16 h and RT-PCR analysis was performed to assess astrocyte activation. All data are mean ± SEM (*n* = 4) with *p ≤ 0.05, **p ≤ 0.01, ***p ≤ 0.001. (TIFF 819 kb)

Additional file 4: ELISA of astrocyte culture supernatants. Concentration of CCL2, CCL20, and IL-6 was measured in the culture supernatants of astrocytes cultured in medium, Th1 and Th17 supernatants. Since we did not remove T cells factors, to exclude any background resulting from T cells, supernatants used in these experiments to stimulate astrocytes were also tested for these factors. CCL2 and IL-6 was not detected in T cell supernatants whereas little amount of CCL20 was detected in Th17 supernatants. Actual concentration of CCL20 released by astrocytes was calculated by subtracting the concentrations obtained for Th17 supernatants. The results are mean ± SEM (*n* = 4) with *p ≤ 0.05 and **p ≤ 0.01. (TIFF 216 kb)

Additional file 5: Inhibition of reactive astrocytosis affects Th17 infiltration into the CNS. Expression of *Rorc* mRNA was measured in the spinal cord of wild-type (WT) or GFAP TK (Tg) mice with established EAE where at day 7 following onset of clinical symptoms the mice were either challenge with PBS (controls) or ganciclovir (GCV) to deplete astrocytes. The basal expression was adjusted to WT-PBS controls and fold changes in *Rorc* mRNA expression was determined for the other groups. Each point represents data from one mouse. Here we used Mann-Whitney test. (TIFF 276 kb)

## Abbreviations

BBB: Blood-brain barrier
BDNF: Brain-derived neurotrophic factor
BMEC: Brain microvascular endothelial cell
CCL: C-C chemokine ligand
CCR: C-C chemokine receptor
CNS: Central nervous system
CNTF: Ciliary neurotrophic factor
CXCL: C-X-C chemokine ligand
EAE: Experimental autoimmune encephalomyelitis
GAPDH: Glyceraldehyde 3-phosphate dehydrogenase
GDNF: Glial cell line-derived neurotrophic factor
GFAP: Glial fibrillary acidic protein
GM-CSF: Granulocyte macrophage colony-stimulating factor
HPRT: Hypoxanthine phosphoribosyltransferase
IFN: Interferon
IGF: Insulin-like growth factor
IL: Interleukin
IP: Intraperitoneal
MFI: Mean fluorescence intensity
MHC: Major histocompatibility class
mRNA: Messenger ribonucleic acid
MS: Multiple sclerosis
NGF: Nerve growth factor
NOS2: Nitric oxide synthase 2
RNA: Ribonucleic acid
RORC: Retinoic acid orphan receptor C
RT-PCR: Reverse transcription polymerase chain reaction
TK: Thymidine kinase
TNF: Tumor necrosis factor
