# Supplementary material for: Effectors of Th1 and Th17 cells act on astrocytes and augment their neuroinflammatory properties
Source: J Neuroinflammation. 2017 Oct 16;14:204. doi: 10.1186/s12974-017-0978-3 (PMC5644084; doi:10.1186/s12974-017-0978-3)
Supplement: Supplementary file 1 — TaqMan probes used for RT-PCR analysis. (DOCX 11 kb) [file 12974_2017_978_MOESM1_ESM.docx]

**Additional file 1:** Taqman probes used for RT-PCR analysis

| **Gene** | **Assay** |
| --- | --- |
| CCL2 | Mm00441242_m1 |
| CCL20 | Mm01268754_m1 |
| CxCL10 | Mm00445235_m1 |
| CxCL12 | Mm00445553_m1 |
| IL-1β | Mm01336189_m1 |
| IL-10 | Mm00439616_m1 |
| IL-6 | Mm00446190_m1 |
| TNF-α | Mm00443258_m1 |
| NOS-2 | Mm00440485_m1 |
| TGF-β1 | Mm00441724_m1 |
| NGF | Mm00443039_m1 |
| BDNF | Mm01334042_m1 |
| GDNF | Mm00439560_m1 |
| IGF-1 | Mm00439560_m1 |
| RORc | Mm01261022_m1 |
| HPRT | Mm00446968_m1 |
| GAPDH | Mm99999915_g1 |
