# Supplementary figures and images for: Effectors of Th1 and Th17 cells act on astrocytes and augment their neuroinflammatory properties
Source: J Neuroinflammation. 2017 Oct 16;14:204. doi: 10.1186/s12974-017-0978-3 (PMC5644084; doi:10.1186/s12974-017-0978-3)

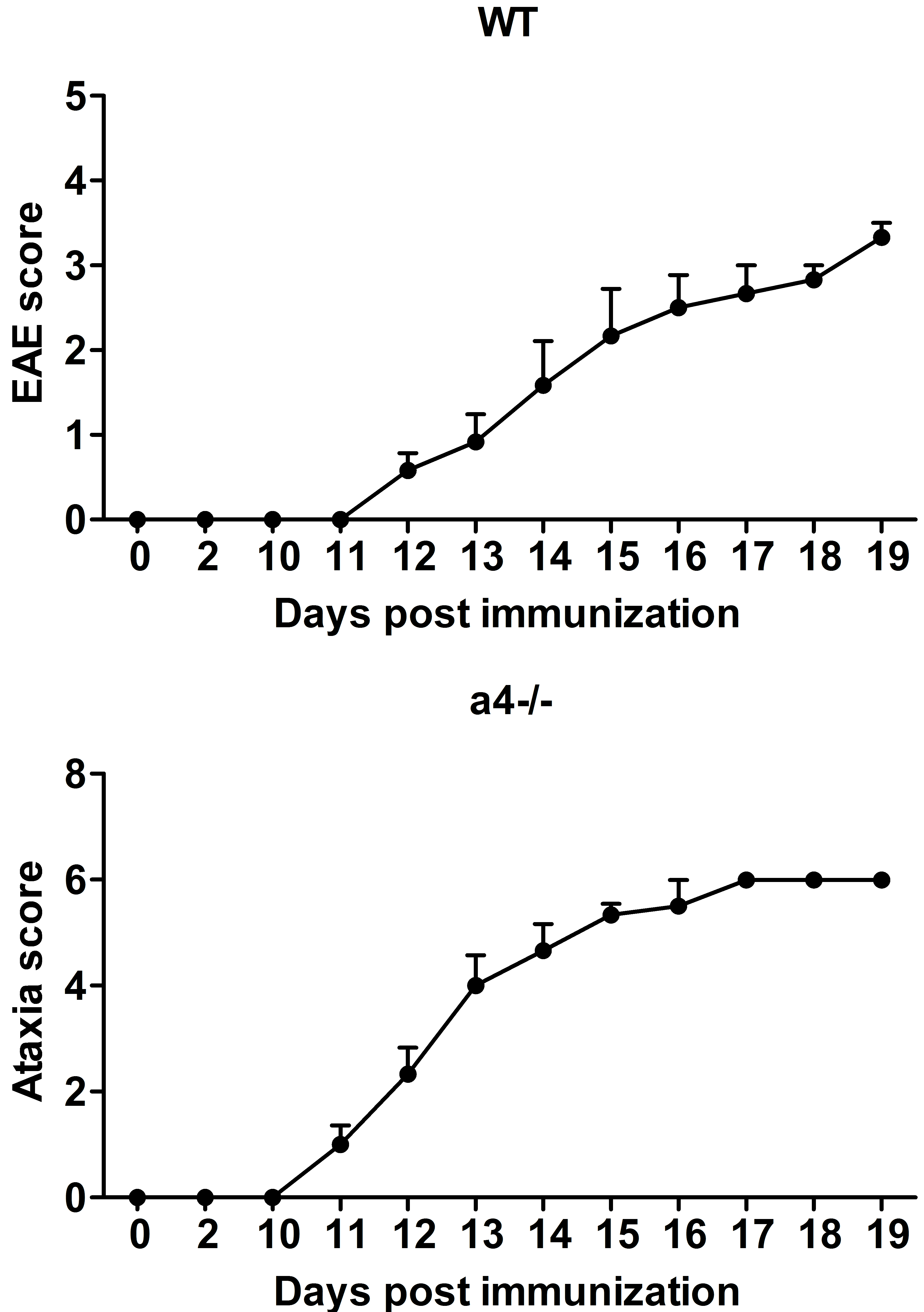

Supplement: Supplementary file 2 — Clinical course of WT and α4−/− mice. EAE severity was monitored in WT and α4−/− mice following immunization with MOG35–55 in CFA. WT mice showed the signs of classical EAE whereas α4−/− mice had an ataxic EAE syndrome. Data is represented as mean clinical score + SEM (n = 6). (TIFF 419 kb) [file 12974_2017_978_MOESM2_ESM.tif]

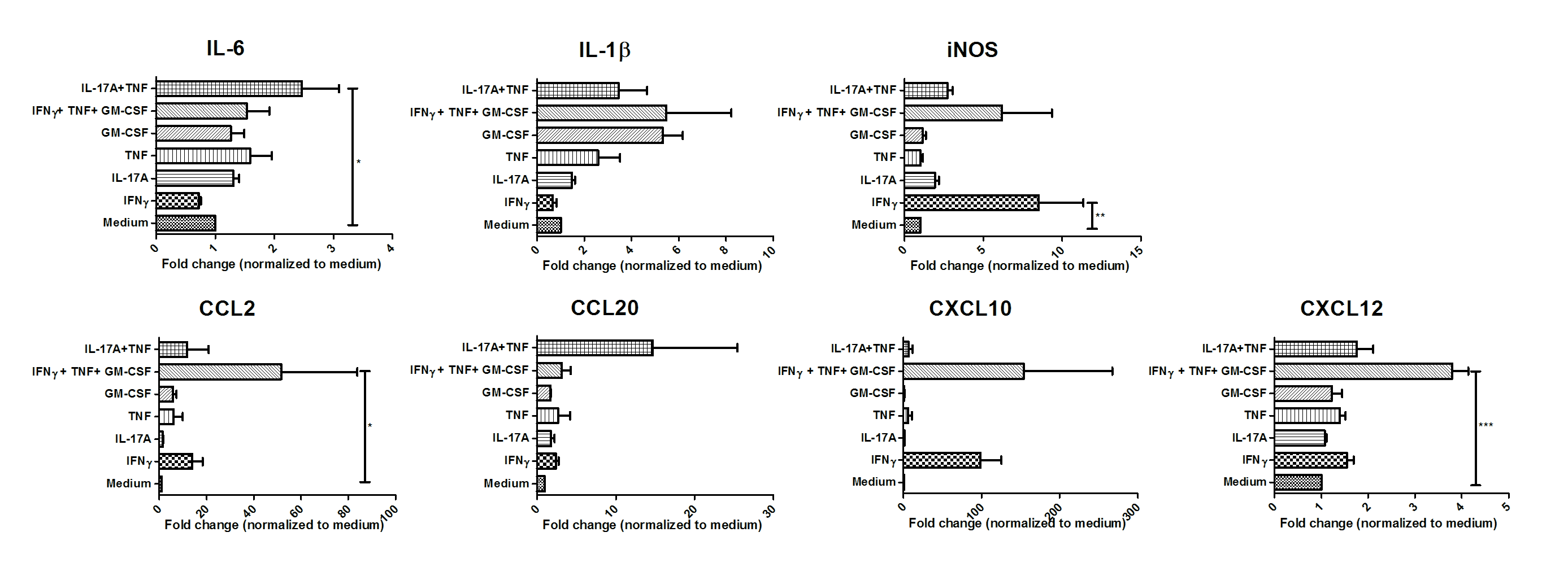

Supplement: Supplementary file 3 — Astrocyte response to effector cytokines. Astrocytes treated with recombinant cytokines for 16 h and RT-PCR analysis was performed to assess astrocyte activation. All data are mean ± SEM (n = 4) with *p ≤ 0.05, **p ≤ 0.01, ***p ≤ 0.001. (TIFF 819 kb) [file 12974_2017_978_MOESM3_ESM.tif]

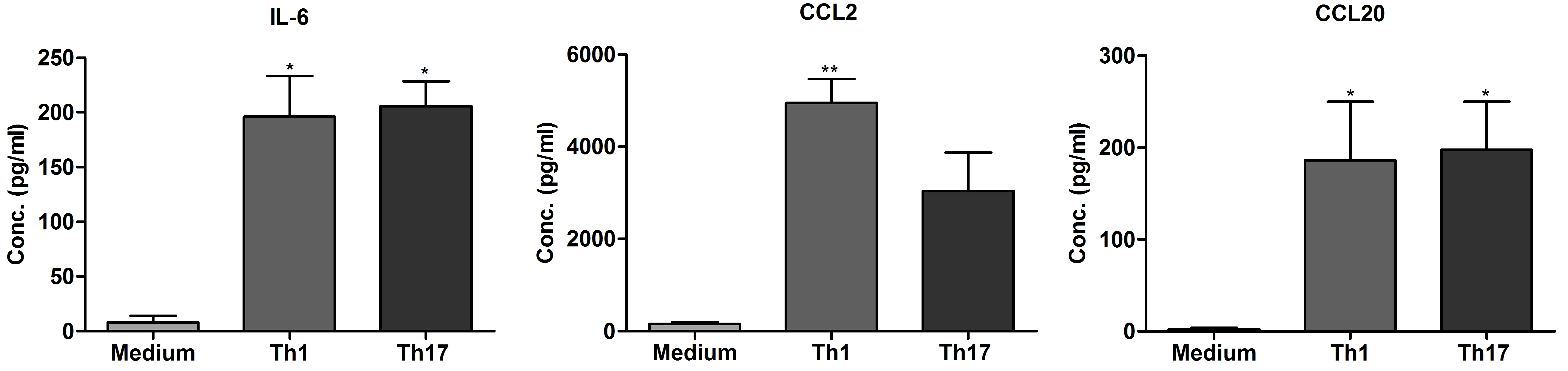

Supplement: Supplementary file 4 — ELISA of astrocyte culture supernatants. Concentration of CCL2, CCL20, and IL-6 was measured in the culture supernatants of astrocytes cultured in medium, Th1 and Th17 supernatants. Since we did not remove T cells factors, to exclude any background resulting from T cells, supernatants used in these experiments to stimulate astrocytes were also tested for these factors. CCL2 and IL-6 was not detected in T cell supernatants whereas little amount of CCL20 was detected in Th17 supernatants. Actual concentration of CCL20 released by astrocytes was calculated by subtracting the concentrations obtained for Th17 supernatants. The results are mean ± SEM (n = 4) with *p ≤ 0.05 and **p ≤ 0.01. (TIFF 216 kb) [file 12974_2017_978_MOESM4_ESM.tif]

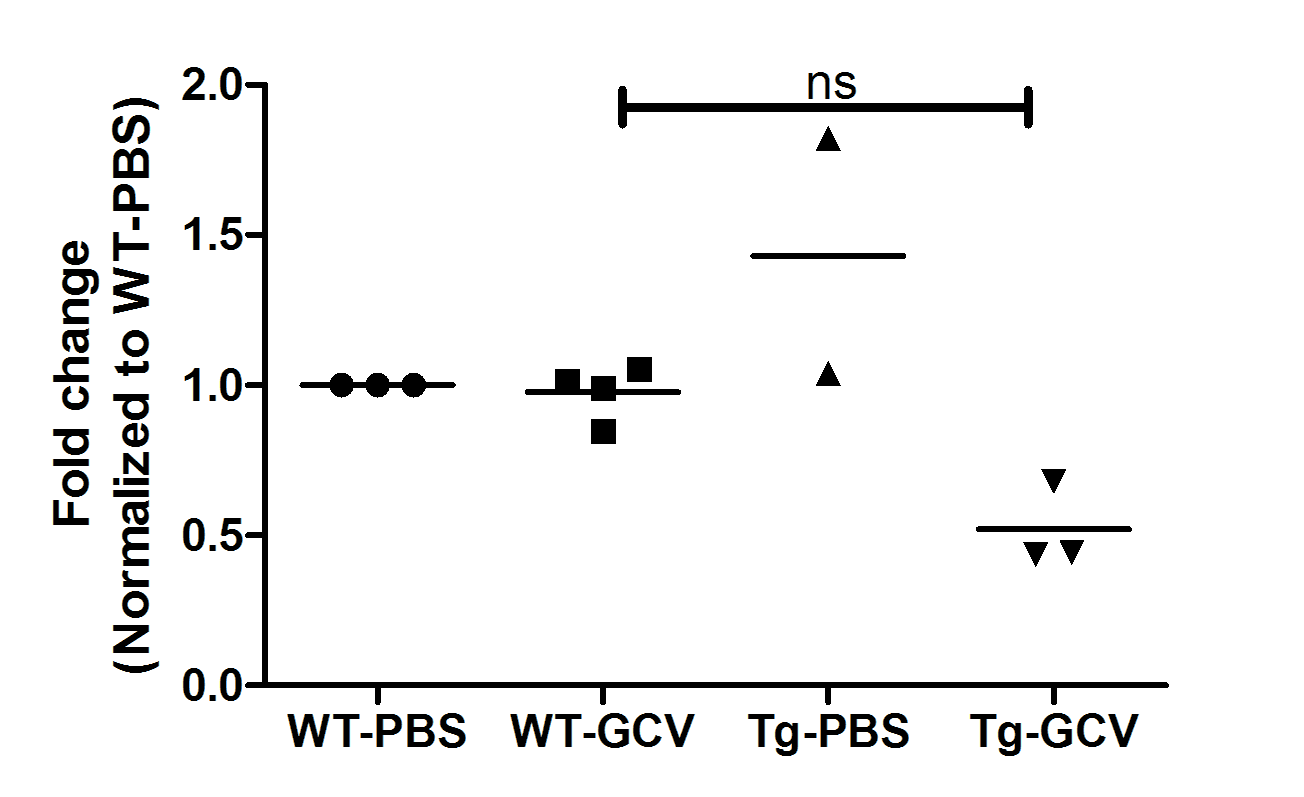

Supplement: Supplementary file 5 — Inhibition of reactive astrocytosis affects Th17 infiltration into the CNS. Expression of Rorc mRNA was measured in the spinal cord of wild-type (WT) or GFAP TK (Tg) mice with established EAE where at day 7 following onset of clinical symptoms the mice were either challenge with PBS (controls) or ganciclovir (GCV) to deplete astrocytes. The basal expression was adjusted to WT-PBS controls and fold changes in Rorc mRNA expression was determined for the other groups. Each point represents data from one mouse. Here we used Mann-Whitney test. (TIFF 276 kb) [file 12974_2017_978_MOESM5_ESM.tif]
